# THE RELATIONSHIP OF SELF-MONITORING OF LIFESTYLES WITH PHYSICAL FUNCTION AND METABOLITES IN DIABETIC OLDER ADULTS

**DOI:** 10.1093/geroni/igad104.2798

**Published:** 2023-12-21

**Authors:** Yan Du, Lixin Song, Mitzi Gonzales, Sara Espinoza, Carlos Jaen, Jing Wang, Kumar Sharma

**Affiliations:** UT Health San Antonio, San Antonio, Texas, United States; University of Texas Health Science Center at San Antonio, San Antonio, Texas, United States; UT Health San Antonio, San Antonio, Texas, United States; Cedars-Sinai Medical Center, Los Angeles, California, United States; UT Health San Antonio, San Antonio, Texas, United States; Florida State University, Tallahassee, Florida, United States; UT Health San Antonio, San Antonio, Texas, United States

## Abstract

Type 2 diabetes (T2D) is a major risk factor for functional decline in older adults. Mobile/wearable devices are evidenced to improve lifestyles for diabetes management and improve functionality. This study assessed the associations of technology tracked lifestyle and self-monitoring behaviors with glycemic control, physical function and mitochondrial function related metabolites in older adults with T2D. Fitbit fitness trackers were used to track physical activity and food intake over 12-week in 15 older adults with T2D (age=70.5±4.8). Physical function was assessed by Short Physical Performance Battery (SPPB) and grip strength. First void urine was used to assess metabolites. The associations of percentage of days with tracked steps (PDWTS), percentage of days with food log (PDWFL), and 7-day mean steps with HbA1c, SPPB, grip strength and metabolites were examined using Pearson Correlation analysis. At week 12, PDWTS was negatively associated with 3-Hydroxyisovaleric acid and Hydroxyhippuric acid (P< 0.05). PDWFL was negatively associated with HbA1c, Hydroxyisovaleric acid, Hydroxyhippuric acid, and Hydroxybutyric acid (p< 0.05); SPPB was not related to any self-monitoring and lifestyle behaviors, but was negatively related to Hydroxyisovaleric acid, Glycolic acid and Hydroxyisobutyric acid (p< 0.05). In older adults with T2D, self-monitoring might improve glycemic control. Self-monitoring and SPPB were related to several urine metabolites, but the underlying mechanisms is unclear in this study with small sample size. Future research with sufficient power is needed to investigate the underlying mechanisms of self-monitoring and lifestyle behaviors on these metabolites, and how these metabolites might be applied to improve physical function in older adults with T2D.

